# Applying Unique Molecular Indices with an Extensive All-in-One Forensic SNP Panel for Improved Genotype Accuracy and Sensitivity

**DOI:** 10.3390/genes14040818

**Published:** 2023-03-29

**Authors:** Adam Staadig, Johannes Hedman, Andreas Tillmar

**Affiliations:** 1 Department of Forensic Genetics and Forensic Toxicology, National Board of Forensic Medicine, 587 58 Linköping, Sweden; 2 Department of Biomedical and Clinical Sciences, Faculty of Medicine and Health Sciences, Linköping University, 582 25 Linköping, Sweden; 3 National Forensic Centre, Swedish Police Authority, 581 94 Linköping, Sweden; 4 Applied Microbiology, Department of Chemistry, Lund University, 221 00 Lund, Sweden

**Keywords:** forensic genetics, unique molecular indices, UMI, massively parallel sequencing, single nucleotide polymorphism, kinship

## Abstract

One of the major challenges in forensic genetics is being able to detect very small amounts of DNA. Massively parallel sequencing (MPS) enables sensitive detection; however, genotype errors may exist and could interfere with the interpretation. Common errors in MPS-based analysis are often induced during PCR or sequencing. Unique molecular indices (UMIs) are short random nucleotide sequences ligated to each template molecule prior to amplification. Applying UMIs can improve the limit of detection by enabling accurate counting of initial template molecules and removal of erroneous data. In this study, we applied the FORCE panel, which includes ~5500 SNPs, with a QIAseq Targeted DNA Custom Panel (Qiagen), including UMIs. Our main objective was to investigate whether UMIs can enhance the sensitivity and accuracy of forensic genotyping and to evaluate the overall assay performance. We analyzed the data both with and without the UMI information, and the results showed that both genotype accuracy and sensitivity were improved when applying UMIs. The results showed very high genotype accuracies (>99%) for both reference DNA and challenging samples, down to 125 pg. To conclude, we show successful assay performance for several forensic applications and improvements in forensic genotyping when applying UMIs.

## 1. Introduction

One of the key challenges within forensic genetics is to increase sensitivity, enabling detection of the smallest possible amounts of DNA. However, several analysis techniques include a certain level of interfering noise, which could hinder the interpretation. To increase the sensitivity, one approach would be to develop techniques that can distinguish true signals from noise; more specifically, from a forensic genetic perspective, to distinguish true alleles from false variants. Massively parallel sequencing (MPS) is one technique that has revolutionized the field of forensic genetics and has been shown to be a powerful method for forensic DNA analysis [1,2,3,4]. However, technical artefacts exist, and when analyzing samples with low template DNA, distinguishing between true and false alleles can be difficult. One of the most common error types is caused during the PCR amplification process. Stutter artifacts are well known when analyzing traditional short tandem repeat (STR) markers. These are caused by strand slippage of the DNA polymerase during the amplification process [5]. The stutter phenomenon is not an issue when analyzing single nucleotide polymorphisms (SNPs) due to the lack of repetitive regions in SNP loci. However, there are other amplification issues, such as polymerase base substitution errors [6], which could result in erroneous PCR products. A misincorporated base early in the cycling process could result in incorrect genotype interpretation. In addition, the risk of amplification errors increases when analyzing low copy numbers of DNA, mainly due to stochastic effects [7]. Another source of error originates from the sequencing process, where base substitutions can occur [8,9].

Unique Molecular Indices (UMIs), also known as unique molecular identifiers or molecular barcodes, were initially introduced as a tool to count the absolute number of molecules [10,11,12,13,14] and were later applied to the field of medical genetics for sensitive detection of cell-free DNA [15,16,17,18,19]. For instance, early detection of circulating tumor DNA is an important strategy for detecting tumor development, determining treatment and monitoring drug response by quantitative measures of circulating cell-free tumor DNA [20]. A UMI is a short random nucleotide sequence, commonly 8–12 base pairs long. These random sequences can either be incorporated into the sample during an initial PCR [18] or enzymatically ligated prior to amplification [15]. Addition of the UMI sequences enables bioinformatic detection of the original template molecules. Since all reads have a UMI attached, one can distinguish reads that result from PCR amplification (i.e., having the same UMI sequence) and reads that represent the original template molecules (i.e., having unique UMI sequences). Errors resulting from both amplification and sequencing can be present in the final reads; however, by counting the number of unique UMIs instead of all reads, the error rates could be reduced. From all reads with the same UMI sequence, a consensus read (or UMI read) is created. If a variant is presented only in some of the reads carrying the same UMI, this variant will be filtered out and considered as a false variant. A variant is considered to be true if most of the reads carrying the same UMI have the variant present, and the specific read number threshold can be user defined. One commercially available kit that has incorporated UMI technology is the QIAseq Targeted DNA Custom Panel (Qiagen, Hilden, Germany). This assay is a multiplexed PCR based on a single primer extension technology. The QIAseq kit was primarily developed and evaluated for the detection of circulating DNA with high amounts of DNA (>10 ng) available [15,21,22]. Even though UMIs have mainly been used in medical applications, the UMI principle could be applied within forensics as well. A sensitive and accurate detection of low-level DNA variants could potentially be a successful technological improvement for the field of forensic genetics. At present, and as far as we know, only a few studies applying UMI from a forensic perspective have been conducted [23,24,25].

The rapid technological advancements of MPS and increased knowledge about the human genome have enabled additional forensic applications of DNA, such as DNA intelligence. For instance, DNA can be used as an investigative lead to narrow down the list of suspects. The prediction of human appearance, such as eye, hair, and skin color, from DNA has been well described [26] as has the prediction of biogeographical ancestry [27]. A more recent adoption in the field is investigative genetic genealogy (IGG), which has generated crucial investigative leads for the identification of unknown perpetrators in a number of criminal cases [28,29] as well as the identification of human remains [30]. An extended DNA profile is required for the IGG method, which preferably consists of hundreds of thousands of SNPs which, for instance, can be generated via high-density SNP microarrays or whole genome sequencing assays. The high-density SNP profile can then be uploaded to a public genealogy database to trace biological relatives to the unknown by matching segments of shared DNA [31,32,33]. Subsequently, the traditional genealogy investigation hopefully results in a candidate suspect, and traditional forensic methods, such as STR typing, are used to either confirm or reject the candidate. The use of STR markers as a confirmation method is feasible as long as the quality of the sample is high enough to enable generation of a sufficiently informative STR profile; however, some forensic casework samples can be heavily degraded, such as old bone samples. The STR typing can, in such cases, result in partial DNA profiles and, in the worst case scenario, insufficient information for identification. Furthermore, if the reference sample is a distant relative, mainly in cases of identifications of historical human remains, the STR markers can be too few to generate a sufficiently high support for any of the tested hypotheses. Due to these limitations in STR typing, a SNP-based approach could be a more appropriate alternative. Recently, extensive marker panels with thousands of SNPs have been developed for forensic applications [34,35,36,37,38].

The FORensic Capture Enrichment (FORCE) panel [34] is an extensive all-in-one SNP marker set for different forensic applications. The panel consists of carefully selected SNP markers, including ancestry, phenotype, identity, and kinship informative markers, as well as X- and Y-chromosomal SNPs. This panel can be applied with different enrichment and sequencing methods based on different chemistries. A hybridization capture technique (myBaits, Arbor Biosciences, Ann Arbor, MI, USA) was used and evaluated in the initial FORCE publication [34]. The main aim of this study is to investigate the potential of UMIs in forensic genetics by applying the UMI technology together with the FORCE panel. We assessed the impact of incorporating UMIs on the genotype accuracy and sensitivity by evaluating the observed genotypes with and without considering the UMIs. Additionally, we evaluated the overall FORCE QIAseq assay performance by analyzing different sample types of forensic relevance, such as mock case, degraded and DNA mixture samples. Furthermore, we investigated the potential of this panel for casework-like applications such as kinship analysis, forensic DNA phenotyping and biogeographical ancestry predictions.

## 2. Materials and Methods

### 2.1. The FORCE Panel

The FORCE panel can be adopted with several enrichment and sequencing strategies. In this study, all samples were analyzed with a QIAseq Targeted DNA Custom Panel (Qiagen) [39] comprising the FORCE SNPs. All DNA libraries were sequenced on a MiSeq FGx instrument (Verogen, San Diego, CA, USA). For this FORCE QIAseq assay, 5507 SNPs were selected. 

### 2.2. Sample Selection

All samples were handled and analyzed in accordance with the ethical approval by the Swedish Ethical Review Authority (Dnr 2022-06781-01). 

#### 2.2.1. Reference Samples

Repeatability, sensitivity and genotype accuracy were investigated based on three different reference samples. Two of the samples (NA12877 and NA12878) were provided by the Coriell Institute for Medical Research (Camden, NJ, USA), and one was 2800M (Promega, Madison, WI, USA). All three samples were analyzed with 20 ng of DNA as input. NA12877 and 2800M were analyzed in duplicate. The five samples were pooled and sequenced together. A dilution series of NA12877 was prepared with the following input amounts of DNA: 10 ng, 1 ng, 0.5 ng, 0.25 ng, 0.125 ng, 0.06 ng, 0.03 ng and 0.015 ng. All eight samples in the dilution series were sequenced together. 

#### 2.2.2. Mixture Samples

The two Coriell samples, NA12877 and NA12878, were mixed in four different ratios, 1:1, 1:10, 1:50 and 1:100, with NA12878 as the major contributor. All mixtures were analyzed in duplicate, with 10 ng DNA as input amount. The ability to detect a mixture was evaluated by investigating the allele read frequency (ARF) distribution and by calculating the heterozygosity rate [40]. The ARF for each locus was calculated by dividing the read depth of the allele with the most reads with the total number of reads. Density plots of the ARF values for both mixture and single-source samples were plotted in R [41] to illustrate the distribution. Additionally, we evaluated the ability to extract the genotypes from one unknown individual in the mixture, assuming genotypes from the other individuals were known. This could represent a true case with a DNA mixture of victim (known genotypes) and perpetrator (unknown genotypes). This was done for the 1:1 mixture, with a quantitative approach [31], by removing the read counts for the known contributor, assuming 50% contribution. The remaining reads were used to determine the genotypes of the unknown contributor. For those reads, we applied a coverage threshold of 10× and an allelic balance threshold for homozygotes of ≥0.90 and for heterozygotes of ≤0.55 for the genotype calling. Subsequently, call rate and accuracy for the extracted genotypes were calculated. 

#### 2.2.3. Mock Case Samples

One female saliva sample was extracted with a Chelex-based extraction method [42]. Two different amounts of DNA (1 ng and 10 ng) were treated with two known PCR inhibitors, soil (humic substances) and moist snuff, which represent two known inhibitors in Swedish forensic casework samples. The soil solution was prepared by mixing soil with nuclease-free water (20% *w*/*w*) and shake-incubated for one hour [43]. Subsequently, 5 µL of the soil solution was added to the initial library preparation step, together with 1 ng and 10 ng DNA. Moist snuff solution was prepared by leaching snuff bags in 1 mL nuclease-free water to extract the inhibitors [43]. Then, 0.6 µL of the supernatant was added to the initial library preparation step, with 1 ng and 10 ng DNA as input. The saliva samples were also analyzed without any inhibitor for comparison. The 10 ng untreated sample was further used as reference when conducting genotype concordance tests with the inhibitor-spiked samples.

#### 2.2.4. Bone and Tissue Samples

Eight human skeletal bone samples were selected and extracted with two different extraction methods: a PrepFiler BTA method with Automate Express (Thermo Fisher Scientific, Waltham, MA, USA) and a phenol/chloroform-based extraction assay [44]. Additionally, four human tissue samples were selected and extracted with a phenol-chloroform based extraction method [44]. All bone and tissue samples had previously been analyzed in case work, generating complete STR profiles. All samples were diluted to 1 ng prior to analysis. Six of the bone samples had previously been analyzed with a forensically validated in-house SNP panel [45] consisting of 131 SNPs overlapping with the FORCE panel. Furthermore, six bone samples had been analyzed with the ForenSeq DNA Signature Prep kit [46] with 167 overlapping SNPs. Thus, concordance rates were calculated between FORCE QIAseq genotypes and the two additional panels. 

#### 2.2.5. Kinship Samples

Kinship-based assessment was performed based on blood samples from two different families with known relations, each consisting of the two parents and their three children, giving a total of 10 samples. DNA was extracted, and 1 ng of DNA was used for the library preparation. Based on the observed DNA data from the kinship informative SNPs (max 3935 SNPs), consistency with Mendelian inheritance patterns was verified, and likelihood ratio (LR) calculations were performed in Familias [47]. Allele frequencies from the SweGen project [48], consisting of allele frequencies of a Swedish population, were used for the LR calculations. Paternity tests for each of the children were calculated in both trio (including known mother, alleged father and child) and duo cases (including alleged father and child). Additionally, maternity tests in duo cases (alleged mother and child) were also performed for all children. 

To further examine the informativeness in paternity duo cases, 1000 simulations were performed in Familias with the following hypotheses: H1: The alleged father is the biological father of the child; H2: The alleged father is unrelated to the child. The number of genetic inconsistencies were counted when hypothesis H2 was simulated as the true hypothesis. 

To assess the informative power of the panel for more distant relationships, ranging from second to fifth degree relatives, simulations were performed 1000 times for each of the following hypotheses. The simulations were performed in ILIR [49] based on allele frequencies from a Swedish population generated from the SweGen project [48]. Genetic linkage was accounted for using genetic position information from a Rutgers map [50].

- 2nd degree relation: Half siblings (H1) versus unrelated (H2)
- 3rd degree relation: First cousins (H1) versus unrelated (H2)
- 4th degree relation: First cousins once removed (H1) versus unrelated (H2)
- 5th degree relation: Second cousins (H1) versus unrelated (H2)

#### 2.2.6. Phenotype and Ancestry Predictions

Phenotype and ancestry predictions were performed for two individuals (blood samples) based on the phenotype and ancestry informative SNP markers. Eye, hair and skin color predictions were done with the HIrisPlex-S web tool [51,52,53], and FORCE QIAseq generated genotypes were converted to HIrisPlex-S compatible nomenclature. The results were compared with self-reported eye, hair and skin color information for the tested individuals. Biogeographical ancestry predictions were performed using FamLink2 [54,55] with a naïve Bayes–based approach. Reference samples comprised allele frequencies for the autosomal SNPs from seven meta populations (African, American, East Asian, European, Middle Eastern, Oceanic and South Asian). The self-reported ancestry for the two individuals was reported as country of origin for their grandparents. See Supplementary Table S1.

### 2.3. Library Preparation

The library preparation was performed with the QIAseq Targeted DNA Custom Panel (Qiagen), consisting of the FORCE SNPs. All samples were analyzed according to the manufacturer’s recommendations specified in the protocol [39]. All samples were quantified prior to library preparation using the Qubit 2.0 fluorometer (Thermo Fisher Scientific). The initial step of the library preparation was a multienzymatic reaction consisting of fragmentation, end-repair and A-addition. This was immediately followed by an adaptor ligation step, which included ligation of both the sample-specific index and the UMIs. The adapter-ligated DNA were then cleaned twice with QIAseq magnetic beads. Target enrichment was then performed using single primer extension of the specific targets in a PCR reaction, according to the protocol of 6 cycles of 15 s at 98 °C and 15 min at 65 °C. The target enrichment was followed by a second QIAseq magnetic bead-based clean-up and a universal PCR, including ligation of the second sample specific index. The cycling conditions followed the manufacturer’s protocol, and the number of cycles was set to 19. The second PCR was followed by a final QIAseq magnetic bead-based clean-up. The final libraries were then quantified using the Qubit 2.0 fluorometer. The DNA integrity was checked using the High Sensitivity DNA kit on the 2100 Bioanalyzer (Agilent Technologies, Santa Clara, CA, USA). The samples were diluted to 4 nM based on the quantification and fragment size distribution of the samples. Samples were then pooled, denatured and further diluted to 10 pM, which was loaded onto the MiSeq FGx (Verogen) instrument. Additionally, a QIAseq A Read 1 Custom Primer I was loaded according to the manufacturer’s protocol, and a paired-end 2 × 151 bp sequencing was selected. The number of samples pooled for sequencing varied from three to eight per sequencing; this is described in more detail in Supplementary Table S2. 

### 2.4. Bioinformatic Analysis with UMI

The bioinformatic workflow was built in the CLC Genomics Workbench V.21.0.3 (Qiagen). All thresholds and settings were set to default. The resulting FASTQ files from the MiSeq FGx were imported into CLC, and the initial step was the *Remove and annotate with unique molecular indices* tool. The UMI sequences, together with the common sequence, were removed to improve the efficiency and accuracy of the read mapping. The reads were then annotated with the UMI information for further analysis. This was followed by a read mapping with hg19 as the reference genome with the *Map reads to reference* tool. All the mapped reads that also belonged to the same UMI were annotated with a UMI group ID with the *Calculate Unique Molecular Index Groups* tool. Based on these groups, a single consensus read was created (UMI read) using the *Create UMI reads from grouped reads* tool. These UMI reads were then aligned to the same position as the original read. This was followed by *Remove ligation artifacts* to reduce erroneous reads that originated from the adaptor ligation step. Next, the *InDels and structural variants* tool was used to identify structural variants, relying on information from unaligned ends. This information was then used for a second alignment, the *Local realignment*, which is used to improve the initial read mapping. *Identify known mutations from read mapping* was used to identify the reads at the specific SNP positions. The final step was then to annotate the identified variants with the UMI information by using the tool *Annotate variants with UMI info.* Final genotype calling was performed in Microsoft Excel with a defined coverage threshold at 10×. The ARF threshold for homozygotes was set to ≥0.95, and for heterozygotes to ≤0.80. The quality score threshold was set to ≥15. 

### 2.5. Bioinformatic Analysis without UMI

One approach to evaluate the power of UMI is to analyze the same sequencing data without taking the UMI information into consideration. We analyzed the same sequencing data by counting the total number of reads, including the PCR duplicates, which is the traditional bioinformatic workflow when evaluating MPS data, thus ignoring the UMI information. This was done in CLC Genomics Workbench V.21.0.3 by importing the same FASTQ files as above. The first step was to use the *Remove and annotate UMI information* tool to remove the UMI and thereby improve the read mapping. Secondly, the reads were mapped to the reference genome (hg19) with the *Map reads to reference* reads tool. This was followed by the *InDels and structural variants* tool to identify structural insertions and deletions from the mapping. Next, a *Local Realignment* was performed to further improve the read mapping, and finally the *Identify known mutations from read mapping* was used to identify the reads at each specific locus. The resulting read counts were then analyzed in Microsoft Excel for genotype calling. This approach was applied to the dilution series of the Coriell sample NA12877.

The ARF and quality score thresholds for the non-UMI data were the same as for the UMI approach above. However, the coverage thresholds for the non-UMI data varied and were set with two different approaches. Firstly, the coverage for the UMI data was set to 10×, and the non-UMI data coverage was set so that the call rates between the two data sets were similar (i.e., increasing the coverage threshold for the non-UMI data). From this, error rates were compared between the UMI and non-UMI data. Secondly, the coverage for the non-UMI data was adjusted so that the error rates were similar with and without UMI information. From this approach, the call rates between the UMI and non-UMI data sets were compared. The hypothesis was that the use of UMI increases sensitivity and genotype accuracy. This implies that if the call rates are similar, the error rates would be lower in the UMI data. In addition, if the error rates between the two data sets are similar, the call rate would be higher in the UMI data. 

## 3. Results

In total, 5507 SNPs were selected to be included in the panel. Six markers were excluded during the primer design. One reason for exclusion was that the genome context close to the SNP region was not unique. The erroneous region could be amplified and, subsequently, false variants could be detected since the reads could map in multiple places in the genome. Another reason was that the genome context close to the SNP had either an abnormally high or low GC % content or extremely repetitive regions, resulting in difficulties in designing specific and efficient primers. Therefore, the primer design resulted in primers for 5501 SNPs. Most of the SNPs had two primers covering the SNP region, which would reduce the impact of population-specific nucleotide polymorphism in the primer site. A distribution of the distance from primer to SNP is illustrated as a histogram in Supplementary Figure S1. Additionally, four SNP markers were excluded, since no read data was observed at these sites for any of the analyzed samples. Thus, 5497 SNPs markers were further evaluated. See Supplementary Table S3 for a detailed description of all the included SNPs. In total, 58 samples were analyzed on 15 sequencing runs. Supplementary Table S2 shows the DNA input amount, average coverage and FASTQ file sizes for all samples. Additionally, sequencing quality metrics are presented. 

### 3.1. The Effect of Applying UMIs

**Figure 1:**
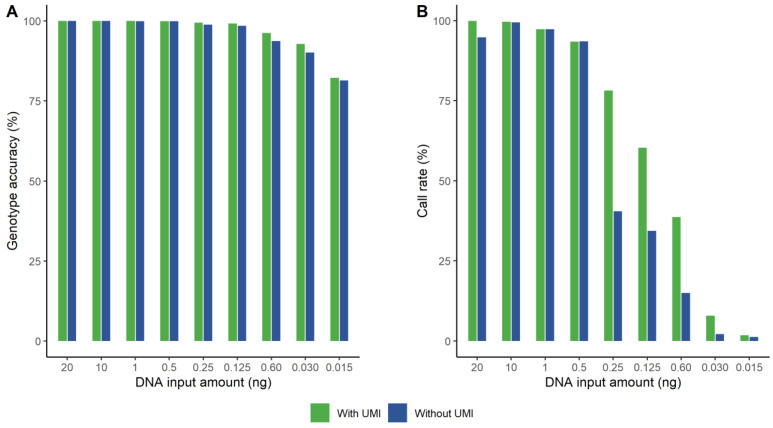
The effect on genotype accuracy (**A**) and call rate (**B**) for different amounts of DNA when applying unique molecular indices (UMIs) (green) compared to without taking the UMI information into account (blue). (**A**) Genotype accuracy when both data sets have similar call rates. The genotype accuracy was significantly higher (*p* < 0.05) for the UMI data, especially for 0.25 ng and lower. (**B**) Call rates when both datasets have similar genotype accuracy. The call rate was significantly (*p* < 0.047) higher for the UMI data, as visually notable for 0.25 ng and lower.

Nine samples with various concentrations of NA12877 were bioinformatically analyzed both with and without taking the UMI information into account. We applied two different approaches to evaluate the data by defining different coverage thresholds, since the number of reads varies when counting UMI reads compared to counting all reads. When applying a threshold resulting in similar call rates between the two datasets, the genotype accuracy increased when taking the UMIs into account. This is illustrated in Figure 1A. The genotype accuracy was similar down to 500 pg, though it was always slightly higher with UMI. For 250 pg and lower, the difference is visually notable. A pairwise *t*-test showed that the difference was statistically significant (*p* < 0.05). The other approach was to set a threshold that resulted in similar error rates and then compare the call rates (Figure 1B). With the same genotype accuracy for the two data sets, the call rates were always higher when taking UMIs into account, especially for the lower DNA amounts. The difference was statistically significant (*p* < 0.047), applying a pairwise *t*-test. The 1 ng sample without UMI did not reach the same high genotype accuracy as for the data with UMI information, regardless of coverage threshold. We decided to plot equal call rates even though the genotype accuracy was slightly lower for the non-UMI data.

### 3.2. General Assay Performance

#### 3.2.1. Genotype Accuracy and Repeatability

The Coriell sample NA12877 was analyzed in duplicate (labeled NA12877-1 and NA12877-2), with 20 ng of DNA as input. The genotype accuracy was assessed by comparing the generated genotypes for each of the duplicate samples with previously published genotypes for NA12877. In the first sample (NA12877-1), complete genotype accuracy was seen for the 5490 called SNPs. Seven markers (0.13%) (rs7537605, rs1710456, rs4092077, rs1428142, rs367600495, rs576471146 and rs169250) were not typed due to imbalance in both heterozygote and homozygote genotypes. For the replicate sample (NA12877-2), six markers (0.11%) (rs1710456, rs4092077, rs1428142, rs710160, rs367600495 and rs169250) resulted in no calls due to the same reason. One discordant genotype (0.02%) was observed in the NA21877-2 sample as an allele drop-out in marker rs7537605. The same marker was inconclusive in the NA12877-1 sample due to imbalance. The number of called genotypes in both replicates were 5489 (99.8%), and complete concordance between the samples was observed. 

The NA12878 reference is a female sample; therefore, 4610 markers were evaluated (excluding the Y-SNPs). Complete genotype accuracy was found in the 4601 called markers. Nine SNPs (0.20%) were not typed (rs4027132, rs4092077, rs1428142, rs1029047, rs1223550, rs7117433, rs1126809, rs10892689 and rs710160) due to imbalances.

Control sample 2800M was analyzed in duplicate, and the genotypes were compared. Complete concordance was seen for the 5487 SNPs that were called in both replicates. Eight markers (0.15%) (rs4092077, rs1428142, rs1029047, rs200332530, rs372687543, rs367600495, rs9785702 and rs2032672) were not called in both duplicates due to imbalance. Additionally, rs576471146 was inconclusive in one of the duplicates, and rs710160 was inconclusive in the other duplicate. We also compared the FORCE genotypes of 2800M with previously published genotypes from the ForenSeq DNA Signature prep kit [46]. Out of the 169 SNPs analyzed in both assays, complete concordance was seen in both replicates. FORCE genotypes generated with the myBaits assay for 2800M were previously published in [34]. A total of 5386 markers overlapped with the two duplicate samples, and discordance was noticed in three markers (rs7537605, rs169250 and rs9785659).

In total, 19 markers (0.35%) were found to be either inconclusive, due to imbalance, or discordant based on the initial analysis of the three high-quantity reference DNA samples, totaling five samples, including the replicates. Table 1 summarizes all these SNPs, and detailed read data is presented in Supplementary Table S4. Possible reasons for the imbalances and discordances were found for 10 of the markers by examining the regions in the Integrative Genomics Viewer (IGV) software version 2.7.2 [56]. For instance, seven SNPs had polynucleotide stretches close to the SNP site, one locus had a SNP variant in the covering primer region and one SNP mapped to multiple places in the genome. See Supplementary Figure S2 for a detailed description of the observations in IGV.

#### 3.2.2. Sensitivity

**Figure 2:**
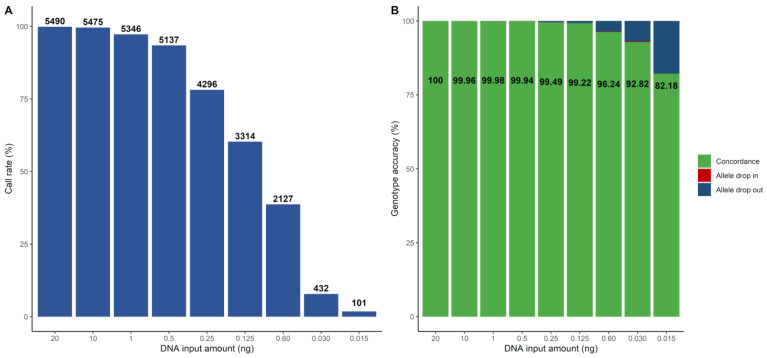
The call rate and genotype accuracy for different amounts of DNA for the NA12877 sample, illustrated as bar plots. (**A**) Call rate, with the number of called SNPs highlighted in text above each bar. (**B**) Genotype accuracy for each sample. The concordance is presented in green and in text. The discordant SNPs are divided into allele drop-ins (red) and allele drop-outs (blue).

The investigation of sensitivity was performed based on the dilution series of NA12877 with the following input amounts of DNA: 20 ng, 10 ng, 1 ng, 0.5 ng, 0.25 ng, 0.125 ng, 0.06 ng, 0.03 ng and 0.015 ng. The call rate was greater than 97% down to 1 ng (Figure 2A). Genotype accuracy greater than 99.9% for the 5497 SNP markers was seen down to 500 pg of DNA input (Figure 2B). In total, four markers were causing the discordances in the samples down to 500 pg, and all of them belonged to the problematic SNPs identified in Section 3.2.1. Thus, if excluding these poorly performing SNPs, complete genotype accuracy was seen down to 500 pg. In addition, genotype accuracy larger than 99% was seen down to 125 pg. Lower amounts of DNA resulted in lower call rates (less than 40%) and, subsequently, the genotype accuracy dropped from 96% at 60 pg to 82% at 15 pg. A substantial majority of the observed discordances from 250 pg and lower were allele drop-outs. One approach to improve the call rates would be to adjust the ARF thresholds to be more non-conservative. We decreased the homozygous ARF value to 0.9 and increased the heterozygous ARF value to 0.85, which resulted in improved call rates. However, a slightly negative effect on the genotype accuracy was observed (Supplementary Figures S3 and S4).

### 3.3. Performance with Casework-Relevant Samples

#### 3.3.1. Mixture Detection and Deconvolution

**Figure 3:**
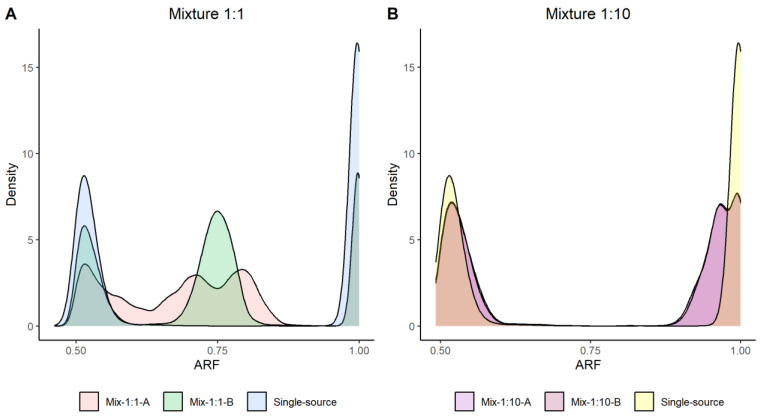
The allele read frequency (ARF) distributions for two different ratios of DNA mixtures, illustrated as a density plot. (**A**) ARF values in two 1:1 mixture samples (red and green) together with a single-source sample (blue). The figure clearly illustrates differences in ARF distribution between the single-source and mixture samples. (**B**) ARF distribution of two 1:10 mixture samples (purple and magenta) together with a single-source sample (yellow); a small shift in the ARF distribution can be seen. There is an overlap in the ARF distribution between the mixture and single-source samples, and the overlap of the colors results in a light-brown color.

Two-person mixtures were analyzed in four different ratios; 1:1, 1:10, 1:50 and 1:100. The aims of the mixture analysis were to firstly detect the mixture, by distinguishing it from a single-source sample, and secondly perform accurate genotype calling for one unknown contributor. Allele read frequencies (ARFs) were calculated for each SNP marker. Differences in the ARF distribution were used to distinguish the mixtures from single-source samples. Figure 3 displays density plots of the 1:1 and 1:10 mixtures together with an ARF distribution for a single-source sample as reference. The 1:1 mixture could clearly be separated from a single-source sample based on the ARF distribution. A more homogenous distribution was seen in the 1:10 mixture compared to a single-source sample. However, a difference was observed, especially as a shift to the left of the ARF distribution for the homozygotes. The two additional mixtures (1:50 and 1:100) could not be distinguished from a single-source sample based on the ARF values (Supplementary Figure S5). Furthermore, an increased heterozygosity rate indicates the presence of a DNA mixture [37]. The heterozygosity rates for two single-source samples and for the mixture samples are illustrated in Supplementary Figure S6 together with the theoretical heterozygosity rate for the investigated mixture. The 1:1 and 1:10 mixture could be detected based on an increased heterozygosity rate. However, the 1:50 and 1:100 mixture could not be detected. 

We performed a mixture deconvolution test for the 1:1 mixture. We assumed a 50% contribution and removed reads that theoretically originated from the known contributor. The remaining UMI reads were used for genotype calling, and the call rates were 59.4% and 82.0% respectively for the duplicates. The genotype accuracy of the called genotypes for the duplicates was 99.2% and 99.9%, respectively, when applying adjusted ARF thresholds (homozygous ≥ 0.90 and heterozygous ≤ 0.55). The discordances were caused by allele drop-ins.

#### 3.3.2. Mock Case Samples

One female saliva sample was analyzed with two different input amounts of DNA, 10 ng and 1 ng. The sample was analyzed with and without the addition of two inhibitors, soil and snuff. The untreated 10 ng sample was used as reference, and concordances were investigated between the inhibitor-spiked samples. Six out of 4610 markers were not called due to imbalance in the 10 ng reference sample; three of those markers were identified as problematic in Section 3.2.1. Complete concordance was seen in both inhibitor-treated samples with 10 ng of DNA. With the 1 ng samples, the call rate dropped to 93%, 90% and 90% for the reference, soil and snuff samples, respectively. The number of discordances were 6 (0.13%), 7 (0.15%) and 14 (0.30%) for the reference, soil and snuff samples, respectively. All discordances were caused by allele drop-outs. The results are summarized in Supplementary Table S5.

#### 3.3.3. Bone and Tissue Samples

DNA from eight bone samples and four tissue samples was analyzed, with 1 ng as input. The call rates ranged from 88% to 99% (Supplementary Figure S7). Six of the bone samples were previously analyzed with a forensically validated in-house SNP panel [45] with 131 SNPs. Complete genotype concordance was seen for all the overlapping SNPs. Additionally, six bone samples were analyzed with the ForenSeq DNA Signature Prep kit (Verogen), and complete genotype concordance with overlapping SNPs (max 167 SNPs) was observed; see Supplementary Table S6.

### 3.4. Forensic Casework Applications

#### 3.4.1. Kinship Analysis

Likelihood ratio (LR) calculations and Mendelian inheritance pattern analyses were performed in the two families with known relations, based on the DNA data from the kinship informative SNPs. Paternity tests were performed in both duo and trio cases, and maternity tests were performed as duos. The compared hypotheses were that each parent is a parent of the child (H1) versus that the parent and child are unrelated (H2). The LR ranged from 6 × 10^263^ to 2 × 10^291^ for the duo cases. The LRs in the trio cases were all above 10^300^. See Supplementary Table S7 for details. One genetic inconsistency (0.002%) was observed between the mother and one child in one of the families, and thus no LR could be calculated without accounting for genotype errors or mutations in the statistical calculation. SNP marker rs7537605 was typed as homozygous AA in the mother and homozygous GG in the child. This marker was found to be problematic in several of the reference DNA samples (Table 1); if excluding this marker, the LR was calculated to 7 × 10^285^. 

**Figure 4:**
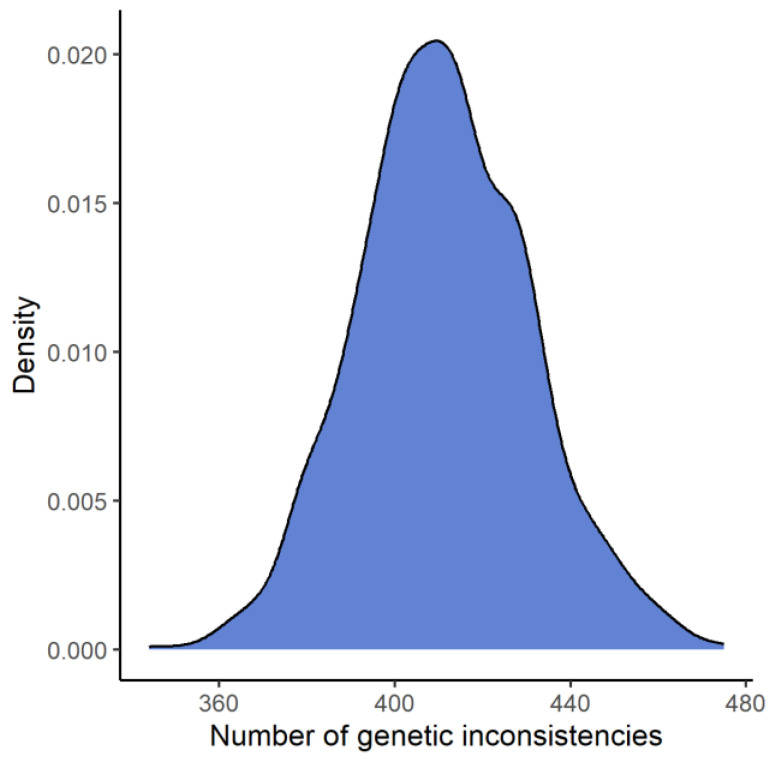
A density plot of the number of genetic inconsistencies from 1000 simulations of a paternity duo case, where the alleged father is unrelated to the child. On average, 411 genetic inconsistencies were observed in the simulations.

Based on allele frequencies for the FORCE kinship informative SNPs from the SweGen project, 1000 simulations were performed in Familias, with the hypothesis that an alleged father is father to the child (H1) versus that the alleged father is unrelated to the child (H2). The number of genetic inconsistencies when the alternative hypothesis (H2) is true was, on average, 411 and is illustrated in Figure 4. The lowest number of genetic inconsistencies was 344 and was observed in one simulation. 

**Figure 5:**
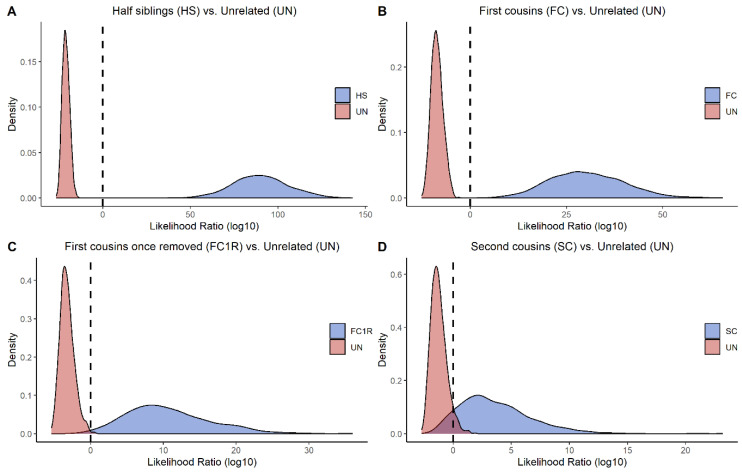
Results from pairwise kinship simulations are illustrated as density plots with four different kinships, all with unrelated as the alternative hypothesis. (**A**) Half siblings (2nd degree); (**B**) first cousins (3rd degree); (**C**) first cousins once removed (4th degree); (**D**) second cousins (5th degree). The dashed line represents the strict definition of likelihood ratio (LR), where LR > 1 represents data in favor of the true relation, and LR < 1 represents data in favor of the hypothesis that tested individuals are unrelated. The different hypotheses are well separated in (**A**,**B**). The two hypotheses in (**C**) can also be separated quite well. The majority of the simulated cases in (**D**) can also be separated, although there are some overlaps of the distribution curves.

Additionally, 1000 simulations were performed in ILIR [49] for evaluating the power of the panel in more distant relationships. The tested hypotheses were two individuals being half siblings, first cousins, first cousins once removed, and second cousins, all with unrelated as the alternative hypothesis. Figure 5 displays a density plot with LRs for each hypothesis. The tested and alternative hypotheses are well separated for the second to fourth degree of relation. For second cousins, the majority of the LRs were still informative; however, some overlap of the LR distribution curves exists. These findings are in concordance with previous results based on allele frequencies from a European population [34].

#### 3.4.2. Phenotype and Ancestry Predictions

Supplementary Table S1 summarizes the phenotype and ancestry predictions for the two samples based on the observed genotypes. All included phenotype and ancestry informative markers were called (44 and 255 SNPs, respectively) in both samples. All predictions were consistent with the self-reported data, except for one sample where the self-reported eye color was intermediate, and the most probable predicted eye color was blue (prediction probability 0.93).

## 4. Discussion

In this study, we evaluated the FORCE panel, which includes ~5500 SNPs, with a QIAseq Targeted DNA Custom Panel (Qiagen), including UMIs. One of the main aims of this study was to explore the power of UMIs in MPS-based genotyping from a forensic genetic perspective. We approached this by analyzing the same raw sequencing data with two different bioinformatic workflows, with and without taking the UMI information into account. We showed that the call rate increased with the UMIs while maintaining the same genotype accuracy. The differences were mainly observed for the lower amounts of DNA, from 0.250 ng, where approximately twice as many genotypes were called. Likewise, the genotype accuracy increased with the UMI information when the call rates were similar in the two data sets. The positive effect on the genotype accuracy was also mainly observed for the lower amounts of DNA. Woerner and Crysup et al. [23,25] previously applied UMIs in a forensic context, focusing on STRs. Consistent with our findings, their results demonstrated that incorporating UMI reads led to improved genotype calling. Additionally, they showed that implementing a machine learning approach for the genotype calling further enhanced the potential power of UMIs. Instead of applying thresholds based on only counting the number of UMI reads, additional parameters were analyzed with the machine learning approach, for instance, the number of reads per UMI read and accounting for possible PCR or sequencing errors in the UMI sequence. Further optimizations and analyses of our bioinformatic workflow would be necessary to evaluate if a similar positive effect on genotype calling could be observed in our data as well. 

The call rates for the five reference DNA samples in the initial analysis were very high (>99.8%); most importantly, the genotype accuracy was >99.9% for all samples. The investigation of sensitivity showed that the call rate started to drop around 1 ng of DNA (97.25% call rate). Although, since the total number of SNPs in this panel is high (~5500), even a low call rate at 60% still represents >3200 SNPs, which, depending on the context, still could be sufficiently informative in many forensic investigations. The concordance of the called genotypes remained very high, greater than 99.9%, down to 500 pg DNA. Furthermore, complete accuracy for the observed genotypes down to 500 pg could be achieved if excluding four of the problematic SNPs (rs4092077, rs1428142, rs7537605 and rs1710456) identified in Section 3.2.1. Similar performance regarding genotype call rate and accuracy was shown by Peck et al. [37] in their validation study of the ForenSeq Kintelligence kit (Verogen), which also is an extensive SNP panel based on multiplex PCR technology. However, we observed a slightly improved genotype accuracy, which could potentially be an effect of the use of UMIs in our data. 

Overall, the assay showed good tolerance for challenging forensic samples, including degraded bone and tissue samples as well as inhibitor-spiked samples. The same type of inhibitors were evaluated in another MPS-based SNP assay, and our results are in consensus with previously published data [57]. It is notable that this assay is more sensitive to the amount of DNA rather than the tested inhibitors, since genotype dropouts increased due to lower amounts of DNA rather than the presence of inhibitors.

A detailed investigation of the problematic SNPs in IGV identified potential complex regions in 10 markers ( Supplementary Figure S2). Seven of the SNPs were located close to a complex polynucleotide region, thus causing difficulties in sequencing or alignment of the reads [8,58]. We observed that some of the problematic SNPs had a considerably high number of “other” nucleotide reads (i.e., not A, C, G or T) ( Supplementary Table S4). This can occur if two different nucleotides are detected on the read 1 (R1) and read 2 (R2) SNP sites. Differences in R1 and R2 could be caused by these polynucleotide regions, thus explaining the observed errors. These 10 problematic SNPs could be excluded in future FORCE panel designs. We could not find any potential reason for the remaining nine problematic markers. However, these were only identified in one sample (or in both replicates of one sample). Three of the 19 identified markers (rs169250, rs1428142 and rs1223550) had previously been reported as poorly performing SNPs in [34]. However, some problematic SNPs identified in this study displayed good performance with the FORCE MyBaits assay, and vice versa. The performance of the SNPs is therefore assay-dependent and should be evaluated separately for each enrichment strategy.

Although the overall call rate is relatively high, at least for DNA quantities down to 1 ng, there can be specific needs to increase the call rate even further. One approach to increase the call rate would be to adjust the ARF thresholds to allow more genotypes to be called. We show in Supplementary Figures S3 and S4 that a more liberal ARF threshold increases the call rates. However, this has a slightly negative effect on the genotype accuracy, since a more generous threshold allows skewed alleles to be typed, which could increase the error rate. It is therefore important that each laboratory defines their needs regarding call rates versus accuracies during internal validation. Furthermore, it is also possible to set specific thresholds for specific types of markers, and the laboratory should consider the application of the data when defining the optimal threshold. For instance, a slightly higher error rate could be acceptable in DNA intelligence applications compared to direct matching or kinship inference. 

Following library preparation, an unexpected 200 bp PCR product was observed in fragment analysis in all DNA libraries with less than 20 ng input DNA. The expected DNA library fragments ranged from 300–600 bp. Supplementary Figure S8 illustrates Bioanalyzer figures of three DNA libraries. This unwanted PCR product could be caused by adapter dimer formation. Too low amounts of DNA would cause the adapters to form dimers instead of binding to the DNA fragments; the number of dimers increased with decreased DNA, as expected. Furthermore, the number of undetermined reads (reads that cannot be assigned to a specific sample) correlated with the number of dimers, implying that the dimers consist of flow cell-compatible adaptor sequences. We noticed that a vast majority of the undetermined reads consisted of a specific DNA sequence originating from read 2 (AACTCCATCAATCAGGTCAGTTTCTCACTTTCAAAACGCAATACTGTACATT) with a specific adaptor sequence (CCAGTCGT). Truelsen et al. [24] noticed a similar phenomenon with dimers for low template DNA samples. They diluted the adaptor indices to adjust the number of adaptors when analyzing low levels of DNA, which successfully decreased the adaptor dimers. However, we applied the same approach and performed a 10-fold dilution of the adaptors without any notable decrease in adaptor dimers. Possibly, additional dilutions could be required, although a too-extensive dilution could have a negative effect by reducing the number of adaptor-ligated reads. Another approach to reduce the adaptor dimer is to repeat the final magnetic bead-based clean-up (Supplementary Figure S8). However, it is quite labor intensive if several clean-ups are required. The QIAseq assay was primarily developed for non-forensic applications, and several studies, with access to high amounts of DNA, have shown successful results [15,21,22]. Still, forensic samples can often have much lower quantities of DNA, and a general assay optimization for low level DNA could be preferable.

We investigated if the low call rate for the 60 pg sample could be caused by the high amount of dimers, which theoretically would decrease the sequencing capacity. The short length of the dimers favors them to cluster more efficiently to the flow cell compared to the intended libraries. Additional magnetic bead-based clean-up was performed five times for the 60 pg sample to reduce the amount of dimers. This sample was then sequenced alone. The resulting call rate was, however, not improved. This indicates that the dimer sequences did not have a negative impact on the number of reads for the 60 pg sample, and the low call rate could be explained by the low amount of input DNA. However, we still believe that the dimer sequences could have a negative effect on the read counts for the intended libraries for samples with higher DNA amount. 

Different strategies can be applied for mixture detection with MPS-based biallelic SNP assays. Mixtures can be identified by observing variation in the allele read frequency or by detecting an increase in heterozygotes [40]. We could successfully distinguish mixtures from single-source samples down to 1:10 mixtures by applying either of the proposed detection methods. The additional analyzed DNA mixtures (1:50 and 1:100) could not be visually distinguished from single-source samples; however, improved depth of coverage could potentially enable more sensitive mixture detection. Previous studies of MPS-based assays have shown similar detection limits [37,40]. However, the heterozygosity rate could not be used to distinguish the ratio of the mixture, i.e., differentiate a 1:1 mixture from a 1:10 mixture. The ARF distribution plot, illustrated in Figure 3, elegantly separates the two different mixture ratios. This could be important for downstream analysis and mixture deconvolution. Furthermore, we applied a quantitative model, described in [31], to deconvolute the mixture by extracting reads contributed by a known donor. If, based on the ARF distribution, we assume that the mixture is 1:1, we would extract 50% of the reads. The resulting reads should, theoretically, originate from the unknown donor. We observed that we needed to adjust the thresholds to be more conservative for mixture deconvolution compared to single-source genotype calling. The accuracy of the deconvoluted genotypes was >99.2% for both replicates. Moreover, the call rate was as low as 59.4% for one of the duplicate samples. However, due to the high number of SNPs in the FORCE panel, that proportion of markers still represents more than 3200 SNPs, which likely would be sufficient for direct human identification or close kinship cases. The results are only based on one duplicate sample, and further research is required to find optimal strategies for genotype deconvolution of DNA mixtures from sequencing data.

The 3935 kinship Informative SNPs in the FORCE panel generated considerably high likelihood ratios for both maternity duos and paternity duos and trios. We observed one discordant genotype in one of the families with known relations. The discordant SNP was found to be problematic in several other samples as well and could, preferably, be excluded in future panel designs. Even though we analyzed biallelic SNPs, which are less informative per marker compared to common forensic STR markers, the average number of genetic inconsistencies in a paternity duo case with an unrelated alleged father was 400. This means that we, on average, observed one genetic inconsistency in every 10th kinship SNP. Furthermore, the simulation results of more distant relations presented in this paper showed great potential for predicting relations from second to fifth degree based on allele frequencies of a Swedish population. Our results are consistent with simulation results based on European allele frequencies [34], which was expected. 

The phenotype and ancestry informative markers have previously been identified and found to be informative [53,59,60]. We have shown that all these markers could be successfully recovered with this assay. The predictions were consistent with the self-reported phenotypes and ancestries, except for one eye color prediction. However, the prediction of intermediate eye color has previously been shown to be difficult [51], and the aim of this study was not to evaluate the prediction power but rather to show that we can analyze the phenotype and ancestry informative SNP markers with the FORCE QIAseq assay including UMIs. 

## 5. Conclusions

This study aimed to evaluate the power of unique molecular indices (UMI) in forensic genetic applications and to show the utility of the FORCE panel with a QIAseq Targeted DNA Custom Panel. We showed that both sensitivity and genotype accuracy were improved when taking UMIs into account. The differences were mainly observed for low amounts of DNA. In total, 5497 SNP markers were analyzed, and both very high call rate (>99.8%) and genotype accuracy (>99.9%) were seen for high quality reference samples. Additionally, the assay showed good tolerance for challenging forensic samples, such as bone and tissue samples, as well as inhibitor-spiked samples. A few SNPs displayed poor performance, and we suggest that some of these should be excluded in future designs of the panel. Based on analysis of the dilution series, the call rate started to drop from 1 ng of DNA input (call rate 97.25%). However, complete genotype accuracy was observed down to 500 pg DNA when excluding the four problematic SNPs. DNA mixtures could be detected down to 1:10 mixtures using ARF distributions or heterozygosity rates, and we successfully deconvoluted a 1:1 mixture with >99.2% genotype accuracy for the observed genotypes. Extremely high likelihood ratios (in the range of 6 × 10^263^) were observed for maternity and paternity tests with known relation. In addition, simulations showed that second to fifth degree relationships could be predicted with strong statistical power, applying the kinship informative SNPs. Additionally, phenotype and ancestry informative SNPs were successfully typed. To summarize, we showed that the QIAseq assay of the FORCE panel has great potential for various types of forensic applications. Finally, our results showed an improved genotype accuracy and sensitivity when applying UMIs, and this technological improvement should be further evaluated and ultimately implemented by the forensic community. 

## Figures and Tables

**Table 1:** The 19 markers found to be either inconclusive or discordant in at least one of the five analyzed reference DNA samples, including the type of SNP and observations from analysis in IGV.

rsID	Type of SNP ^1^	Inconclusive in Number of Samples	Discordant in Number of Samples	Observation from Analysis in IGV ^2^
s1428142	Kinship	5	0	Complex polynucleotide region
rs4092077	Kinship	5	0	Imbalance in reference BAM file
rs367600495	Y-SNP	4	0	Complex polynucleotide region
rs1029047	iiSNP	3	0	Complex polynucleotide region
rs710160	Kinship	3	0	Complex polynucleotide region
rs169250	Kinship	2	2	Complex polynucleotide region
rs1710456	Kinship	2	0	Non-specific read mapping
rs200332530	Y-SNP	2	0	-
rs2032672	Y-SNP	2	0	-
rs372687543	Y-SNP	2	0	-
rs576471146	Y-SNP	2	0	Complex polynucleotide region
rs7537605	Kinship	2	2	SNP in primer site
rs9785702	Y-SNP	2	0	-
rs10892689	Kinship	1	0	Complex polynucleotide region
rs1126809	piSNP	1	0	-
rs1223550	Kinship	1	0	-
rs4027132	Kinship	1	0	-
rs7117433	Kinship	1	0	-
rs9785659	Y-SNP	0	2	-

^1^ Kinship-informative, Y-chromosome-informative (Y-SNP), identity-informative (iiSNP) and phenotype-informative (piSNP) SNP. ^2^ Observation from analysis in integrative genomics viewer (IGV) for possible reasons for the poor performance.

## Data Availability

Data are stored at the National Board of Forensic Medicine, Linköping, Sweden, and may be made available to approved laboratories upon written request.

## Supplementary Materials

The following supporting information can be downloaded at: https://www.mdpi.com/article/10.3390/genes14040818/s1, Figure S1: The distance in base pairs from primer to SNP; Figure S2: Observations from analysis in IGV for ten of the problematic SNPs; Figure S3: Call rates for the dilution series with adjusted ARF thresholds; Figure S4: Concordance for the dilution series with adjusted ARF thresholds; Figure S5: ARF distribution for the 1:50 and 1:100 mixtures; Figure S6: Heterozygosity rate for two single-source samples, expected mixtures and for the analyzed mixture samples; Figure S7: Call rate for bone and tissue samples; Figure S8: Bioanalyzer results of three DNA libraries; Table S1: Phenotype and biogeographical ancestry predictions together with self-reported data; Table S2: Run metrics for all samples and each sequencing run; Table S3: All 5507 included SNPs; Table S4: Problematic SNPs identified in the initial analysis; Table S5: Call rate and concordance for mock case samples; Table S6: Concordance for bone and tissue samples with two other assays; Table S7: Likelihood ratios for maternity and paternity cases with the number of called kinship SNPs for each sample.
